# Effect of recipient-donor sex and weight mismatch on graft survival after deceased donor renal transplantation

**DOI:** 10.1371/journal.pone.0214048

**Published:** 2019-03-29

**Authors:** Frank-Peter Tillmann, Ivo Quack, Magdalena Woznowski, Lars Christian Rump

**Affiliations:** Klinik für Nephrologie, Heinrich Heine Universität Düsseldorf, Düsseldorf, Germany; Medizinische Universitat Graz, AUSTRIA

## Abstract

This study evaluated the combined effect of recipient-to-donor weight and sex mismatch after deceased-donor renal transplantation in a German transplant cohort and the evolution of recipient-to-donor weight difference over a 13-year observation period. The association of absolute weight and sex difference with graft failure was explored in an outpatient cohort of deceased-donor transplant recipients who underwent kidney transplantation between 2000 and 2012. Graft failure was defined as repeated need for dialysis or death with a functioning graft. Recipient and donor sex pairings were classified as sex concordant (MDMR/FDFR) or discordant (MDFR/FDMR). These classes were further stratified into four groups according to recipient-to-donor weight mismatch ≥10 kg (recipient > donor) or <10 kg (recipient < donor). Multivariable Cox proportional hazards models were applied to evaluate the time to graft loss adjusting for donor, immunologic, surgical, organizational, and recipient predictors. Sex-concordant transplant pairings <10 kg weight difference served as the reference group. Among 826 transplant recipients, 154 developed graft failure (18.6%). Median graft survival time was 3.9 years; first quartile (0.2–1.2), second quartile (1.2–2.9), third quartile (2.9–5.8), and fourth quartile (5.8–12.4). After multivariable adjustment, the highest relative hazard for graft failure was observed for sex-discordant transplant pairings with a ≥10 kg weight difference between recipient and donor (compared to the reference group MDMR/FDFR with weight difference <10 kg, MDMR/FDFR with weight difference ≥10 kg, hazard ratio 1.86, 95% confidence interval 1.07–3.32—p = 0.029; MDFR/FDMR with weight difference <10 kg, hazard ratio 1.14, 95% confidence interval 0.78–1.68—p = 0.507, and MDFR/FDMR with weight difference ≥10 kg, hazard ratio 2.00, 95% confidence interval 1.15–3.48—p = 0.014). A recipient-to-donor weight mismatch of ≥10 kg was associated with an increased risk of graft loss or recipient death with a functioning graft. Concurrent sex discordance seemed to enhance this effect as indicated by an increase in the hazard ratio. We detected no significant tendency for increasing recipient-to-donor weight differences from 2000 to 2012.

## Introduction

Renal transplantation has become the primary option for the treatment of end-stage renal disease in many countries. Over the past decades, kidney transplant recipients of organs from deceased donors have benefitted from improvements in survival rates and quality of life compared with end-stage renal disease patients on dialysis [1]. Nevertheless, despite excellent graft survival rates within the first year after transplantation, further improvement of the long-term survival rates of donated organs remains a challenge of major scientific and clinical interest [2]. Numerous studies have identified immunologic and non-immunologic risk factors that contribute to an increased rate of kidney failure or patient death with a functioning graft [3,4]. A number of donor as well as recipient characteristics have been determined to negatively impact graft and patient survival during the early and late periods after transplantation. Among them, donor/recipient size and sex mismatch have been identified as possible risk factors for impaired graft survival. Several studies have focused either on kidney weight [5], body mass index (BMI) [6], or body surface area (BSA) [7] as estimates of transplanted nephron mass after deceased-donor transplantation as well as after living donation [8]. Sex mismatch has also been associated with reduced graft function, but findings on this topic have been inconsistent [9]. The worse outcome seen in female grafts that have been transplanted to male recipients is generally attributed to a size mismatch with resultant nephron underdosing [10], whereas a general immunologic mismatch between the sexes has been assumed due to the minor histocompatibility antigen H-Y [11]. Importantly, these investigations have analysed the effects of weight and sex mis-matches on graft survival as single mathematical variables. Recently, applying a different analytical approach, the combined effect of size and sex mismatch has been explored in a large cohort of deceased-donor transplant recipients using the United States Scientific Registry of Transplant Recipients (SRTR), indicating a higher graft failure rate in cases of concurrent mismatch in donor-recipient weight and sex [12]. Applying the same analytical approach, we aimed at determining the additive effect of weight and sex mismatch after renal transplantation on long-term graft survival in a single center German cohort of deceased-donor transplant recipients. Furthermore, we investigated trends in recipient to donor weight differences over the study period of 13 years.

## Materials and methods

### Study approval

This study was approved by the local ethics committee (Ethikkommission der Medizinischen Fakultät der Heinrich Heine Universität Düsseldorf number 6200R). Data were coded in a manner that ensured subjects could not be identified either directly or through linked identifiers. Since this study involved retrospective review of existing data, Institutional Review Board (local ethics committee) approval was obtained, but without specific informed consent from the patients. All procedures performed in studies involving human participants were in accordance with the ethical standards of the institutional and/or national research committee and with the 1964 Helsinki declaration and its later amendments or comparable ethical standards. The need for informed consent was waived due to the retrospective nature of the research.

### Design

This analysis represents a cohort study of all patients receiving a deceased-donor solitary kidney transplant in our clinic between January 1, 2000 and December 31, 2012. Recipients of organs from living donors, patients younger than 18 years of age, those receiving multiple organs, and those with missing or implausible data were excluded.

### Exposure

We defined the primary exposure as a combination of donor-recipient weight and sex mismatch. Sex pairing between donor and recipient was categorized as either sex-identical female donor to female recipient and male donor to male recipient (FDFR & MDMR) or sex-disparate (FDMR & MDFR). Absolute weight difference between the recipient (R) and the donor (D) was categorized as <10 kg and ≥10 kg (R to D). These absolute weight difference thresholds were recently reported as clinically relevant in a large sentinel investigation of the SRTR [12]. Each recipient-donor sex pairing was subcategorized by absolute weight difference resulting in 4 possible weight and sex pairings. Secondary exposures were sex-pairing (sex-identical versus sex-disparate transplantation) and recipient-to-donor weight mismatch (≥10 kg R to D).

### Outcome

The outcome of interest was graft failure or loss for any reason. Graft failure was defined as the need for chronic dialysis or repeat pre-emptive transplantation or death with a functioning graft.

### Data collection

In addition to the primary exposure, previously reported and presumed predictors of graft loss including donor and recipient height, donor and recipient weight, donor and recipient age, donor and recipient BMI, cold ischemia time, warm ischemia time, dialysis vintage, categories of human leukocyte antigen mismatch (0–6), panel reactive antibody category (0%, 1–19%, ≥20%), cytomegalovirus risk constellation, recipient hepatitis C and B virus status, recipient diabetes mellitus status, and whether transplants were performed under the Eurotransplant Senior Programme were analyzed. All data were extracted from clinical charts or electronic databases, including the hospital's laboratory database and the Eurotransplant's electronic resource (www.**eurotransplant**.org).

### Analysis

We used descriptive statistics to evaluate baseline patient characteristics. Continuous variables were classified by means and SDs or medians and interquartile ranges. Baseline donor and recipient characteristics and the proportion of patients in each sex match/mismatch category were calculated for all patients in both weight categories. The association between donor-recipient sex and weight mismatch and graft failure was analyzed using a multivariable Cox proportional hazards model adjusting for known predictors of graft failure as detailed above. Sex-identical transplant pairs with <10 kg absolute weight difference were defined as the reference group. Relative hazards and 95% confidence intervals (CIs) were graphically displayed for each donor-recipient sex/weight pairing compared with the reference group. We further performed unadjusted and adjusted multivariable Cox proportional hazards models to test for an association of donor-to-recipient weight and donor-to-recipient sex difference with graft loss (secondary analyses). Finally, we searched for an effect of BSA on the primary outcome. Here, two categories of BSA were analyzed in two different approaches, < vs. ≥0.01 m^2^ and < vs. ≥0.20 m^2^ donor-to-recipient difference in BSA, to distribute the cohort into comparably sized categories. Values of p < 0.05 were considered statistically significant. Statistical analyses were performed using SPSS version 20.0, IBM Corp., Armonk, NY.

## Results

### Study population

**Fig 1:**
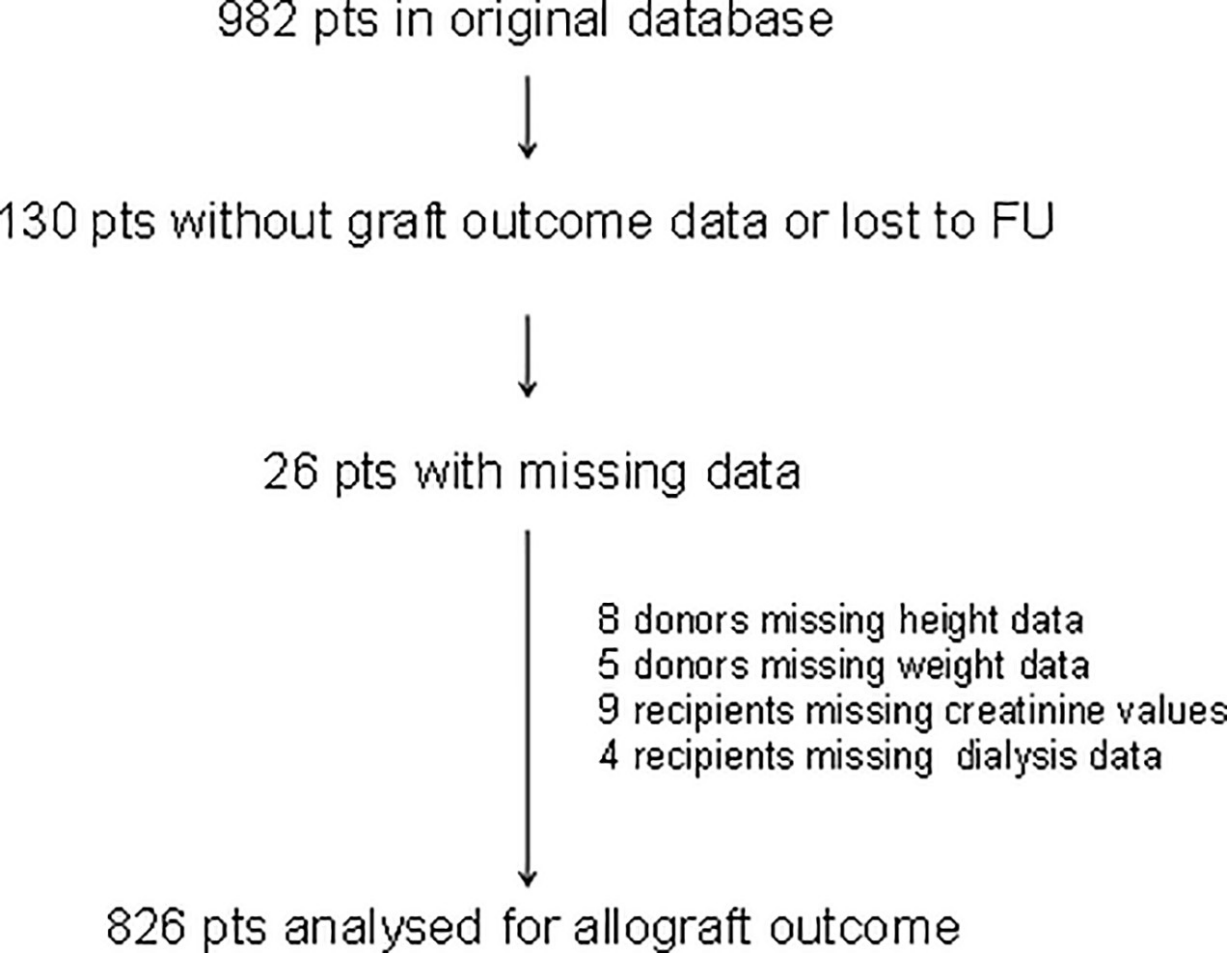
Flow-chart patient eligibility criteria. Eligible for inclusion in our dataset were all deceased-donor solitary kidney transplant recipients with complete data between January 1, 2000 and December 31, 2012.

The initial cohort consisted of 982 deceased-donor renal transplant recipients. We excluded 130 donors due to missing graft outcome data, and 26 were excluded due to incomplete datasets. Therefore, we analyzed 826 complete datasets (**Fig 1**). Among these recipients, 23.5% were ≥10 kg larger than the donors, and 49.5% of the transplants were performed using a sex-discordant graft. Mean recipient weight was 68.7 ± 11.9 kg, and mean donor weight was 82.9 ± 11.4. Mean and median absolute weight difference between the recipient and the donor were -5.4 kg, and -6.0 kg, respectively. Females accounted for 40.3% of the recipients and 48.4% of the donors. Additional baseline characteristics stratified according to sex and weight pairing are shown in **Table 1.**

**Table 1:** Baseline study population characteristics.

Characteristics in numbers (%)	Categories	
	≥ 10 kg (R>D)	<10 kg (R>D)
	n = 194 (23.5)	n = 632 (76.5)
**Donor factors**		
SCD	153 (78.9)	507 (80.2)
ESP	41 (21.1)	125 (19.8)
DCD	0 (0)	0 (0)
Mean age ± SD, yr	52 ± 19	54 ± 15
Sex (male)	67 (34.5)	359 (56.8)
Mean height ± SD, cm	167 ± 10	175 ± 9
Mean donor weight, kg	67 ± 13	83 ± 14
Mean BMI, kg/m^2^	24 ± 4	27 ± 5
**Recipient factors**		
Mean age ± SD, yr	55 ± 11	54 ± 13
Sex (male)	155	338
Mean height ± SD, cm	177 ± 10	169 ± 9
Mean recipient weight, kg	90 ± 14	69 ± 12
Mean BMI, kg/m^2^	29 ± 6	24 ± 4
Diabetes (NIDDM & IDDM)	60 (30.9)	141 (22.3)
Previous kidney transplant	21 (10.8)	110 (17.4)
Mean dialysis vintage ± SD, yr	5.9 ± 3.1	6.3 ± 3.1
Dialysis vintage > 4, yr	141 (72.7)	477 (75.5)
HCV positive recipient	11 (5.7)	35 (5.5)
Mean creatinine at last follow-up, mg/dl	2.76 ± 2.32	2.35 ± 1.84
**Surgical and immunological factors**		
Mean cold ischemia time, h	15.7 ± 5.7	15.8 ± 5.6
Mean warm ischemia time, min	30 ± 14	30 ± 10
Mean peak PRA ± SD	3.4 ± 15.5	3.6 ± 13.7
Peak PRA of zero	177 (91.2)	538 (85.1)
Peak PRA of 1–19	8 (4.2)	58 (9.2)
Peak PRA of > = 20	9 (4.6)	36 (5.7)
**Donor and recipient factors**		
Mean HLA-MM ± SD	2.6 ± 1.8	2.7 ± 1.6
0 MM	35 (18.1)	90 (14.3)
1 MM	20 (10.3)	54 (8.6)
2 MM	39 (20.1)	138 (21.8)
3 MM	43 (22.2)	162 (25.6)
4 MM	24 (12.4)	107 (16.9)
5 MM	18 (9.2)	51 (8.1)
6 MM	15 (7.7)	30 (4.7)
Absolute weight difference (R weight minus D weight), kg	23.2 ± 12.5	-14.2 ± 15.7
Sex-concordant transplant (MDMR & FDFR)	82 (42.3)	335 (53.0)
Sex-discordant transplant (MDFR & FDMR)	112 (57.7)	297 (47.0)

R>D = recipient heavier than donor, SCD = standard criteria donor, ESP = "Eurotransplant Senior Program", DCD = donation by cardiac death is not performed in Germany, BMI = body mass index, NIDDM & IDDM = non-insulin & insulin dependent diabetes mellitus, HCV = hepatitis C virus, PRA = panel reactive antibody, HLA = human leukocyte antigen, MM = mismatch, MDMR = male donor to male recipient, FDFR = female donor to female recipient, MDFR = male donor to female recipient, FDMR = female donor to male recipient.

### Primary analysis

**Fig 2:**
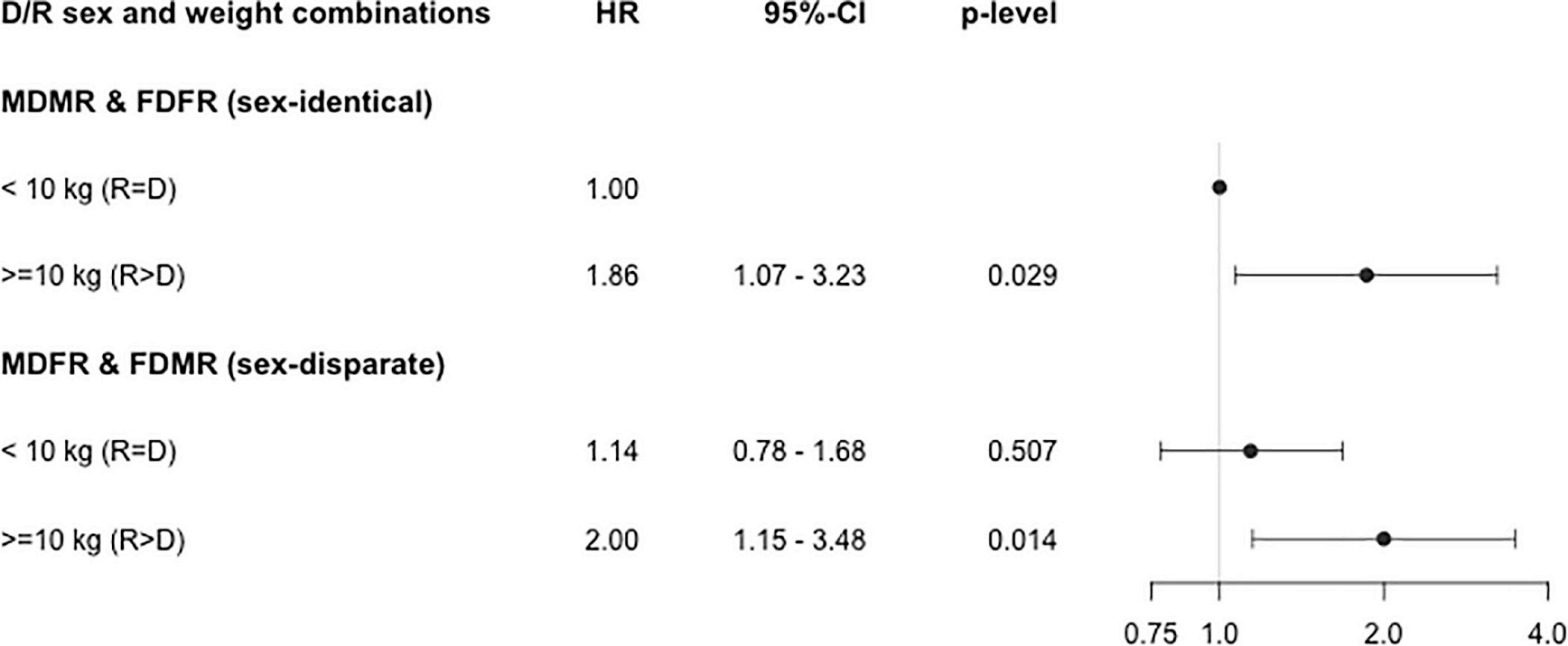
Adjusted Cox-regression analysis and hazards of graft failure. Adjusted Cox-regression analysis showing the hazards of graft failure with corresponding 95% CIs using the following variables: CMV risk constellation of +donor/-recipient, recipient age at transplantation, donor age, recipient body height, donor body height, warm ischemia time, cold ischemia time, dialysis vintage, number of mismatches, peak panel reactive antibody (%) in categories (0, 1–19, ≥20),number of prior transplants, diabetes in the recipient, transplants performed in the "Eurotransplant Senior Programme", and HCV status of the recipient. The risk of graft failure is highest in sex mismatched recipient-donor pairs when the recipient weight is greater than the donor weight. Adjusted relative hazards for graft failure were calculated using the following pairing system: sex-identical transplant (MDMR & FDFR) with weight difference recipient <10 kg than donor (R = D), sex-identical transplant (MDMR & FDFR) with weight difference recipient ≥10 kg than donor (R>D), sex-disparate transplant (MDFR & FDMR) with weight difference recipient <10 kg than donor (R = D), and sex-disparate transplant (MDFR & FDMR) with weight difference recipient ≥10 kg than donor (R>D), MD = male donor, MR = male recipient, FD = female donor, FR = female recipient, 95% CI = 95% confidence interval, R = D recipient weight equal to or smaller than donor weight, R>D recipient weight greater than donor weight, the category sex-identical transplant with a weight difference of <10 kg was used as reference.

**Fig 3:**
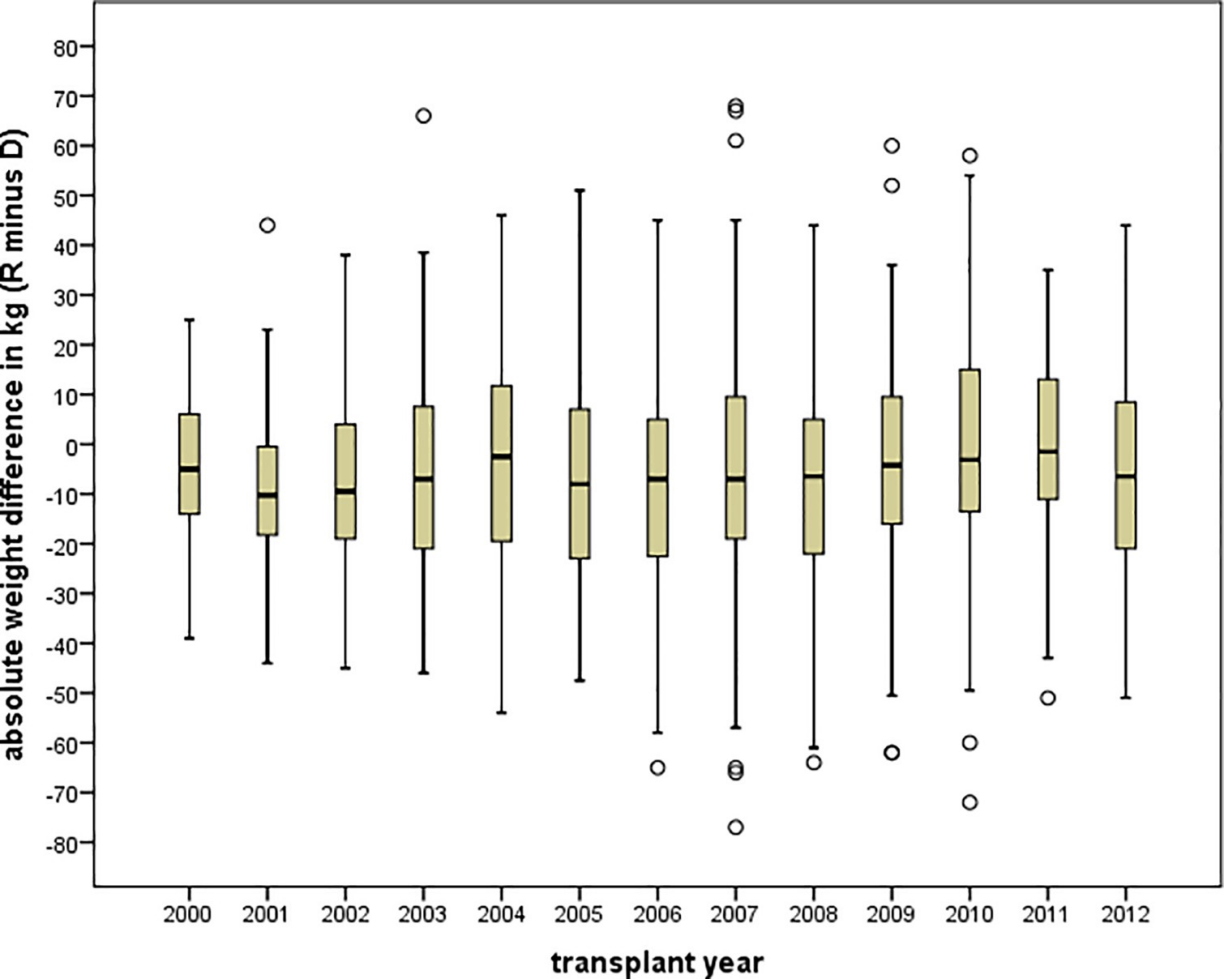
Recipient to donor weight difference per year. Boxplot graphs showing the mean absolute weight difference between recipient and donor in each study year. Although, there was a tendency to increasing interquartile ranges and numbers of outliers, we did not detect a significant increase in recipient-donor weight mis-match in our transplant cohort over an observation period of 13 years. The graphs show lower extreme, lower quartile, median, upper quartile, upper extreme and outliers. R = recipient, D = donor.

Of the 826 individuals included in this analysis, graft failure occurred in 154 patients (18.6%). Mean follow-up time was 3.8 ± 3.0 years and median follow-up time was 2.9 years (interquartile range, 1.2 to 5.8 years). The risk of graft failure for each donor-recipient sex pairing in our cohort was higher when the weight of the recipient was greater than that of the donor. In multivariable Cox regression analysis, the risk of graft loss was highest among recipients of sex-disparate transplants who had a concurrent weight mismatch of ≥10 kg R to D (hazard ration [HR] 2.00, 95% CI 1.15 to 3.48—p = 0.014), relative to sex-identical transplants with no weight mismatch (**Fig 2)**. We further searched for trends in recipient-to-donor absolute weight differences over the study period from 2000 to 2012 (**Fig 3**). Although there was a tendency for enlarged interquartile ranges and increasing numbers of outliers, we did not observe a statistically significant increase in weight difference.

### Secondary analyses

In the unadjusted analysis the risk of graft loss in settings where the recipient's weight was ≥10 kg higher than that of the donor was 1.63 (95% CI 1.15 to 2.30, p = 0.006), whereas the adjusted analysis showed an even greater risk (HR 1.83, 95% CI 1.12 to 2.80, p = 0.005). In contrast to weight mismatch, sex mismatch was not predictive of graft failure in the unadjusted analysis (HR 1.10, 95% CI 0.80 to 1.50, p = 0.574) or the adjusted test (HR 1.15, 95% CI 0.83 to 1.63, p = 0.402). Further details are shown in **Table 2**. Furthermore, we searched also for an effect of sex-disparate transplantation only in patients with a weight-mismatched transplant (R to D weight ≥ 10 kg, n = 194) without yielding significant results on uni- (HR 0.97, 95% CI 0.54 to 1.74, p = 0.925) as well as multivariable Cox-regression analyses (HR 1.14, 95% CI 0.57 to 2,26, p = 0.710).

**Table 2:** Hazard-ratios for graft loss using recipient-donor absolute weight differences and donor-recipient sex concordant and discordant pairing.

Donor-Recipient Pairing	HR (95% CI)	p-value
**Weight-unadjusted**		
<10 kg (R>D)	Ref	
≥10 kg (R>D)	1.63 (1.15 to 2.30)	0.006
**Weight-adjusted**^**a**^		
<10 kg (R>D)	Ref	
≥10 kg (R>D)	1.83 (1.12 to 2.80)	0.005
**Sex-unadjusted**		
MDMR & FDFR	Ref	
MDFR & FDMR	1.10 (0.80 to 1.50)	0.574
**Sex-adjusted**^**b**^		
MDMR & FDFR	Ref	
MDFR & FDMR	1.15 (0.83 to 1.60)	0.402

Statistical analysis adjusted for the following variables: CMV-risk, recipient age at transplant, donor age at transplant, recipient height, donor height, warm ischemia time, cold ischemia time, dialysis vintage, number of human leukocyte antigen mismatches, peak panel reactive antibody, previous kidney transplants, diabetes in the recipient, ESP, and HCV.

HR = hazard ratio, 95% CI = 95% confidence interval, R>D = recipient heavier than donor, MDMR = male donor/male recipient, MDFR = male donor/female recipient, FDFR = female donor/female recipient, FDMR = female donor/male recipient, Ref = reference, CMV-risk = CMV risk constellation with recipient CMV antibody negative and donor CMV antibody positive, ESP = Eurotransplant Senior Programme, HCV = HCV status of the recipient.

^**a**^additionally adjusted for recipient and donor sex.

^**b**^additionally adjusted for recipient and donor weight

### Sensitivity analysis

As BSA has also been reported to predict graft loss, we performed an identical statistical analysis using an exposure variable of combined recipient-to-donor sex and BSA mismatch. Here, two categories of BSA were analyzed with two different approaches; < vs. ≥0.01 m^2^ and < vs. ≥0.20 m^2^ BSA difference from recipient to donor, neither of which yielded significant results on multivariable analysis (data not shown).

## Discussion

A significant impact of recipient factors on donor graft outcome has been observed in the past [13]. Recently, the combined effect of recipient-to-donor weight and sex mismatch has been suggested to represent an independent predictor of graft loss in a large cohort of over 115,000 kidney transplant recipients in the United States [12]. Applying the same analytical approach, our current study aimed at exploring the same effect in a German cohort of transplant recipients. Furthermore, we extended our study approach by evaluating possible trends in recipient-to-donor absolute weight difference over the observation period of 13 years, from 2000 to 2012, in patients attending our outpatient renal transplant clinic. In brief, the results of our investigation are as follows: (I) we also identified an increasing risk of graft failure when the recipient was larger than the donor, (II) this risk seemed to be enhanced in patients with sex-discordant transplants, and (III) we did not detect a significant time-associated increase in absolute weight difference between the recipient and donor over the 13-year study period.

Several studies reported negative graft outcomes in clinical situations involving small kidney donors in relation to the recipient [5,7,14,15]. Generally, it is assumed that body size and other measures of size such as height, weight, and BSA provide both an estimate of the metabolic demand and some indication of the nephron dose, which is determined by the nephron number and glomerular volume. Therefore, it is perhaps surprising that large mismatches in body weight between larger recipients and smaller donors are predictive of decreased graft survival. It has been suggested that consequent nephron underdosing results in hyperfiltration of the remaining nephrons and development of glomerular hypertension with chronic allograft nephropathy and graft failure [16,17]. Nephron formation primarily occurs from the gestational age of 6–36 weeks, and prematurity is a major factor contributing to reduced nephron number [18]. Thus, accumulating evidence has emphasized birth weight as the main predictor of absolute nephron number and body size as a predictor of glomerular volume [19,20]. The present data are fully in line with these hypotheses and prior studies reporting very similar HRs for weight mismatch ≥10 kg when analyzed alone or in combination with sex-concordant transplantation. Although some studies did not find a negative effect of recipient-donor mismatch, these studies involved fewer cases and differed substantially in methodology [21,22].

Sex mismatch has also been reported to potentially contribute to increased sensitization after transplantation, with subsequently reduced graft outcome. It has been postulated that mismatch between the H and Y minor histocompatibility antigens (on the Y chromosome in male donors) promotes allograft rejection or de novo donor-specific antibody evolution with subsequent deterioration of transplant function and graft loss [23,24,25]. In another investigation, the negative impact of a male transplant organ on long-term graft outcome was offset in cases where the donor was larger than the female recipient, supporting both the concept of increased immunological risk in sex-discordant transplant settings and the concept of nephron dosing [6]. Nevertheless, sex mismatch as a predictor of long-term outcome might be confounded by factors inherent to sex-specific behaviours such as medication adherence or life-style, which may be difficult to control for in registry-based investigations [26,27,28]. Unlike the above-mentioned sentinel study [12], we did not detect a significant impact of sex mismatching on graft outcome when sex mismatch was analyzed as a single predictor (i.e., without weight mismatch as a combined predictor) unless the weight difference between recipient and donor was greater than or equal to 10 kg. One major reason for this difference between the two investigations may be the disparities between the study populations. Notably, the large number of cases analyzed by Miller and colleagues enabled the detection of relatively small effects, as indicated by 95% confidence intervals close to one.

Although current techniques in routine clinical practice still do not allow for meaningful nephron counting, promising new techniques are in development [29,30,31]. Hopefully these new techniques may enable researchers to estimate total nephron number more precisely. It will be interesting to observe, if nephron counting has the potential to change allocation policies in the far future. A further promising new technique might be an analysis of metabolomic profiles [32]. This investigation found an association between renal function and altered metabolomic profiles in renal transplant individuals with different degrees of kidney graft function and it will be interesting to see if such altered profiles might also be detectable in sex and weight mismatched transplant cohorts.

Further, we could not identify a massive increase in recipient and donor weight mismatch from 2000 to 2013, although the prevalence of a BMI over 30 in three large German cohorts [33] rose significantly from 1990 to 2011 (in men from 18.9 to 24.5%, and in women from 21.6 to 23.0%). Seemingly, the peak wave of the overweight epidemic has not yet reached our local transplant center as compared to the United States on a nationwide level. Nevertheless, the epidemic might also be foreseeable in Europe. The fact that a clinically relatively low weight difference of 10 kg significantly impact transplant outcome, as indicated by the present results as well as previously published data, calls for a strong combined effort to prevent an overweight epidemic among patients on the waitlist.

There are several limitations to this study. The most important difference to the sentinel investigation by Miller and colleagues [12] lies in the use of a nationwide cohort analysis in the former as compared to a local transplant population at our transplant clinic, with consequently largely reduced patient numbers. Furthermore, the following differences in study design are noteworthy: (I) unlike to the USA, donation after cardiac death is not performed in Germany; (II) the European Senior Transplant Programme does not have a comparable partner program in the United States SRTR; (III) due to the relatively low patient numbers in this cohort, we were not able to use identical categories, instead we built on previously reported evidence and analyzed a reduced set of categories; and (IV) the variable sets applied in the adjusted analyses slightly differed from each other: while we could not account for diabetes mellitus in the donor, we added recipient' HCV status, warm ischemia time, and creatinine on last follow-up to the statistical model. Another major difference between the patient cohorts is the fact that as a result of a relative organ shortage in Germany, the proportion of patients with dialysis vintage times greater than 4 years (70–75%) was almost double than that in the US cohort (35–70%). Furthermore, we could not account for several factors that are generally accepted to negatively impact on graft function and outcome e.g. the development of post-transplant diabetes, the quality of blood pressure control, the recurrence of the primary renal disease in the transplant, recurrent infections of the urinary tract, and aspects of non-adherence. However, in addition to being the first report on the combined effect of weight and sex mismatch in a German cohort, key strengths of our investigation include (I) adoption of the same analytical approach as used in the sentinel investigation and, (II) extension of the model by additional variables possibly impacting the outcome of interest.

## Conclusion

We confirmed the negative impact of recipient-to-donor weight mismatch on graft survival in a German cohort of deceased-donor transplant recipients, and this effect seemed to be enhanced by sex-discordant transplantation. Weight and sex mismatch and their combined effect should be considered in future investigations of long-term graft outcome to elucidate any possible positive and/or negative effects that may be relevant to the implementation of graft allocation systems. Here, expected positive effects on graft longevity must be weighed carefully against possible negative effects on wait times for organ donation in particular patient subgroups.

## Supporting information

S1 File (SAV)
